# P-1973. Characteristics, Clinical Management, and Outcomes of Immunocompromised Patients Diagnosed with COVID-19 in the Outpatient Setting in France

**DOI:** 10.1093/ofid/ofae631.2131

**Published:** 2025-01-29

**Authors:** Essy Mozaffari, Aastha Chandak, Mark Berry, Gaelle Gusto, Giancarlo Pesce, Agnese Restuccia, Michele Bartoletti, Andre Kalil, L O U B E T Paul

**Affiliations:** Gilead Sciences, Foster, California; Certara, New York, New York; Gilead Sciences, Inc., Foster City, California; Certara, New York, New York; Certara Italy, Milano, Lombardia, Italy; Gilead Sciences Europe Ltd, Uxbridge, England, United Kingdom; Department of Biomedical Sciences, Humanitas University, Pieve Emanuele (Italy); IRCCS Humanitas Research Hospital, Rozzano (Italy), Rozzano, Lombardia, Italy; University of Nebraska Medical Center; CHU de Nîmes, Nimes, Languedoc-Roussillon, France

## Abstract

**Background:**

Most COVID-19 patients are treated in primary healthcare settings in France, where patients are typically assigned one general practitioner (GP) for the continuum of their care. The risk of progression to severe disease and death is higher in immunocompromised patients with COVID-19. We descriptively summarize the characteristics, documented clinical management and outcomes of immunocompromised patients diagnosed with COVID-19 in the outpatient setting in France.

**Table 1:**
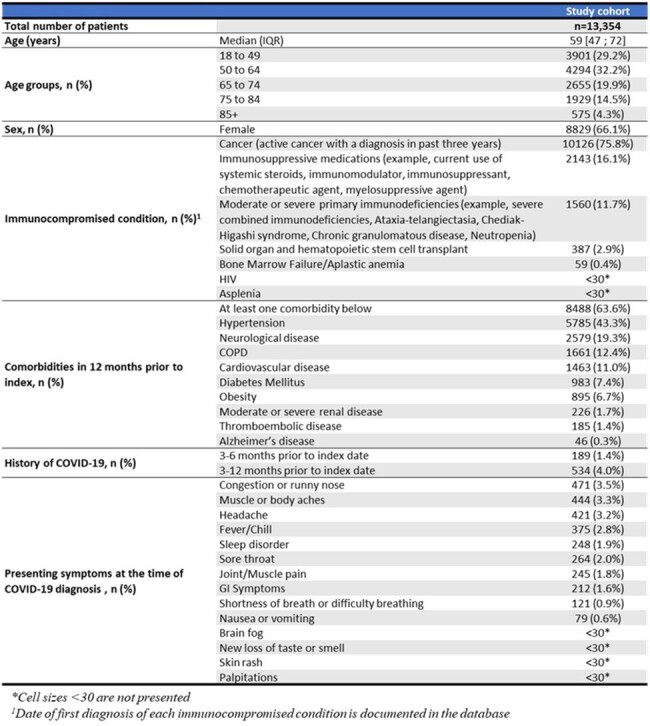
Characteristics of immunocompromised patients diagnosed with COVID-19 in the outpatient setting, 2022-2023

**Methods:**

Adults with an immunocompromised condition and a documented diagnosis of COVID-19 in 2022-2023 were identified from The Health Improvement Network (THIN). THIN collects anonymized patient-level data from electronic health records of GPs and some specialists in the outpatient setting representative of the French population. Index was the first documented COVID-19 diagnosis, and patients with COVID-19 diagnosis the 3-months prior to index were excluded. COVID-19 treatments prescribed within1-month post-index were examined. Reinfection was defined as COVID-19 infection documented in outpatient setting at least 3 months after index.

**Table 2:**
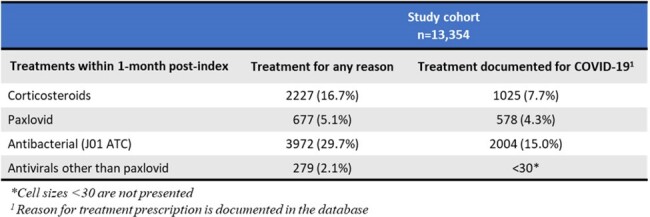
Clinical management of immunocompromised patients diagnosed with COVID-19 in the outpatient setting, 2022-2023

**Results:**

Overall, 13,354 immunocompromised patients with COVID-19 with a median age of 59 years (IQR:47—72), and 66% female were identified. Majority had an immunocompromised condition of active cancer diagnosed in the 3-years prior to index (76%). 64% had at least another key comorbidity documented in the 12-months prior (Table 1).

Treatments prescribed included antibacterials (30%), corticosteroids (17%), nirmatrelvir/ritonavir (5%); COVID-19 was documented as the reason for antibacterial prescriptions (15% overall) and corticosteroids (8% overall) (Table 2).

Post-index, 14% had at least one additional outpatient visit within 7 days, 37% within 30 days, and 54% within 60 days with an average 0.9 (SD=1.1) visits within 60 days (Table 3).

**Table 3:**
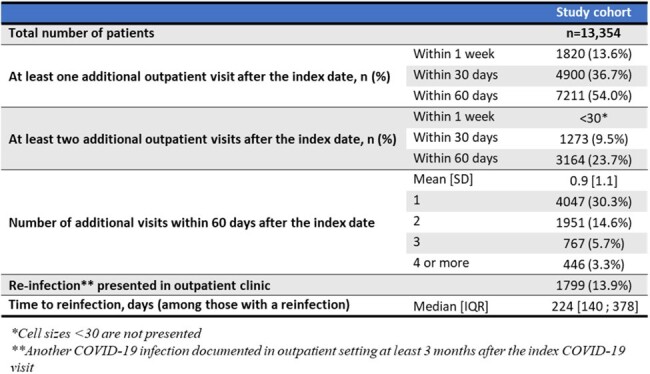
Clinical outcomes of immunocompromised patients diagnosed with COVID-19 in the outpatient setting, 2022-2023

**Conclusion:**

The use of nirmatrelvir/ritonavir was low among immunocompromised patients with COVID-19 diagnosed in outpatient visits. In contrast, inappropriate prescription of corticosteroids and antibacterials was frequent. There is a need for effective treatment options for immunocompromised patients as they face a significant healthcare burden with frequent follow-up visits and COVID-19 reinfections.

**Disclosures:**

Essy Mozaffari, PharmD, MPH, MBA, Gilead Sciences, Inc.: Employee|Gilead Sciences, Inc.: Stocks/Bonds (Public Company) Aastha Chandak, PhD, Gilead Sciences Inc.: My organization (Certara) was contracted by Gilead to conduct this study Mark Berry, PhD, Gilead Sciences, Inc.: Employee|Gilead Sciences, Inc.: Stocks/Bonds (Public Company) Gaelle gusto, PhD, Gilead Sciences Inc.: My organization (Certara) was contracted by Gilead to conduct this study Giancarlo Pesce, Sr Consultant, Gilead Sciences Inc.: My organization (Certara) was contracted by Gilead to conduct this study Agnese Restuccia, PhD, Gilead Sciences: Stocks/Bonds (Private Company) Michele Bartoletti, MD, PhD, Advan pharma: Advisor/Consultant|Advan pharma: Honoraria|Biomereux: Honoraria|Gilead: Advisor/Consultant|Gilead: Honoraria|Infectopharma: Advisor/Consultant|Msd: Advisor/Consultant|Msd: Grant/Research Support|Msd: Honoraria|Pfizer: Honoraria Paul LOUBET, MD, PhD, Astrazeneca: Advisor/Consultant|Gilead: Advisor/Consultant|Moderna: Advisor/Consultant|Pfizer: Advisor/Consultant

